# Supplementary material for: Enantioselective Recognition of L-Lysine by ICT Effect with a Novel Binaphthyl-Based Complex
Source: Micromachines (Basel). 2023 Feb 21;14(3):500. doi: 10.3390/mi14030500 (PMC10056047; doi:10.3390/mi14030500)
Supplement: Supplementary file 1 [file micromachines-14-00500-s001.zip › micromachines-2196396-supplementary.pdf]

Supplementary materials

Shi Tang[1], Zhaoqin Wei[1], Jiani Guo [1], Xiaoxia Sun*[,1] and Yu Hu*[,2]

[1]Jiangxi Key Laboratory of Organic Chemistry, Jiangxi Science and Technology Normal University, Nanchang 330013, China

[2]College of Chemistry, Nanchang University, Nanchang 330031, China

Email: sunxiaoxia77@126.com (X. Sun). huyu@ncu.edu.cn (Y. Hu)

Contents

Supplementary materials 1
Contents 2
Experimental procedures 3
S1、Synthesis and characterization of 3
S1.1 *R*-2,2'-bis(methoxymethoxy)-1,1'-binaphthalene 3
S1.2 *R*-3,3'-bis(hydroxymethylene)-2,2'-bis(methoxy-methoxy)-1,1'-binaphthyl 3
S1.3 *R*-3,3'-bis((prop-2-yn-1-yloxy) methyl)-[1,1'-binaphthalene]-2,2'-diol 4
S1.4 Synthesis of probe *R*-**1** 4
S1.5 Synthesis of probe *S*-**1** 5
S2、^{1}H NMR, ^{13}C NMR and ESI-MS 6
S2.1 ^{1}H NMR, ^{13}C NMR of intermediate products 6
S2.2 ^{1}H NMR, ^{13}C NMR of *R*-**1** 8
S2.3 ^{1}H NMR, ^{13}C NMR of *S*-**1** 9
S2.4 MS-ESI of *R*-**1** 10
S2.5 MS-ESI of *S*-**1** 10
S3、Fluorescence spectra 11
S3.1Fluorescence spectra of *S*-**1** 11
S4、UV-Vis titration 11

Experimental procedures

S1.Synthesis and characterization of

S1.1 *R*-2,2'-bis(methoxymethoxy)-1,1'-binaphthalene

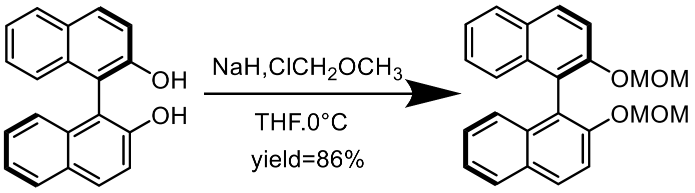

R-a

Under argon protection, sodium hydride (1.22 g, 53.2 mmol) was added to a 500 mL three-necked flask, dissolved with 30 mL of anhydrous THF with stirring. After stirring for 6-8 min under an ice bath, 30 mL of THF dissolved *R*-BINOL (4.0 g, 14.0 mmol) was added slowly dropwise with a constant-pressure dropping funnel. The dropwise addition was completed by stirring until no bubbles emerged. Then chloromethyl organic phase was washed three times with 20 mL saturated sodium chloride solution, dried with anhydrous sodium sulfate, and distilled under reduced pressure to remove the solvent. The resulting crude product was recrystallized with anhydrous methanol to give 4.5 g of white solid in Y=86 % . ^{1}H NMR (400 MHz, $CDCl_3$) δ 8.04 (d, J = 9.0 Hz, 2H), 7.96 (d, J = 8.2 Hz, 2H), 7.67 (d, J = 9.0 Hz, 2H), 7.46–7.40 (m, 2H), 7.34–7.28 (m, 2H), 7.25 (d, J = 8.5 Hz, 2H), 5.17 (d, J = 6.8 Hz, 2H), 5.06 (d, J = 6.8 Hz, 2H), 3.23 (s, 6H).

S1.2 *R*-3,3'-bis(hydroxymethylene)-2,2'-bis(methoxy-methoxy)-1,1'-binaphthyl

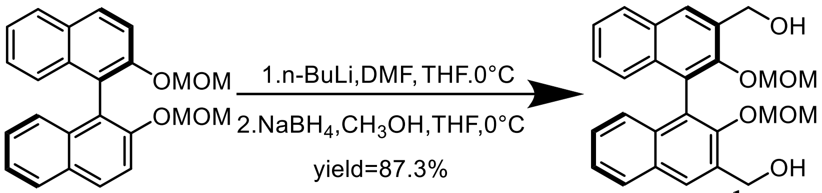

R-a *R*-b

Under the protection of argon, *R*-2,2'-bis (methoxy - methoxy) -1,1'-binaphthyl (4.0 g, 8.1 mmol) was dissolved in 30 mL anhydrous THF in a 250 mL three-necked flask and stirred until completely dissolved, and then stirred for 5 min in an ice bath, and 2.5 M n-butyllithium (2.8 mL, 33.2 mmol) was added slowly dropwise with a constant pressure dropping funnel. The solution was stirred for 2 h and turned yellowish brown. Continue to add anhydrous DMF (2.6 mL, 33.2 mmol) slowly dropwise under the ice bath, and then increase the reaction to room temperature for 3 h after the dropwise addition was completed in five minutes, and after the raw material disappeared by TLC monitoring, add 15 mL of saturated NH_4Cl aqueous solution to quench the reaction and remove the unreacted n-butyllithium, extract with 60 mL ethyl acetate three times, wash the organic phase with saturated NaCl solution three times, dry anhydrous magnesium sulfate. Then The solvent was removed by distillation under reduced pressure to obtain the crude product of 2.5 g of the yellow oily object. The intermediate product (2.5 g, 5.8 mmol) was stirred with 15 mL of THF until it was completely dissolved, then 15 mL of CH_3OH was added and stirred for 10 min in an ice bath, and $NaBH_4$ (0.7 g, 18.6 mmol) was added to the system in batches and stirring was continued until the solution became clarified. The reaction was transferred to room temperature for two hours. After

the raw material disappeared by TLC, the reaction was quenched by adding 10 mL of ice water and then extracted with 30 mL of EA in three batches. The organic layer was washed three times with 20 mL of saturated NaCl solution, dried with anhydrous magnesium sulfate, and distilled under reduced pressure to obtain a yellow-white viscous solid, which was separated and purified by column chromatography (petroleum ether : ethyl acetate =1:1) to obtain a white solid of 2.2 g with a yield of 87.3%. ^{1}H NMR (400 MHz, $CDCl_3$) δ 8.03 (d, J = 11.5 Hz, 2H), 7.97 - 7.82 (m, 4H), 7.43 (t, J = 7.1 Hz, 2H), 7.28 (d, J = 8.3 Hz, 2H), 7.15 (d, J = 8.4 Hz, 2H), 5.07 - 4.90 (m, 4H), 4.46 (q, J = 6.4, 5.9 Hz, 4H), 3.20 (s, 6H). ^{13}C NMR (101 MHz, $CDCl_3$) δ 152.93, 134.64, 133.74, 130.97, 129.46, 128.22, 126.79, 126.13, 125.81, 125.43, 125.17, 99.30, 61.62,57.05. (ppm).

S1.3 *R*-3,3'-bis((prop-2-yn-1-yloxy) methyl)-[1,1'-binaphthalene]-2,2'-diol

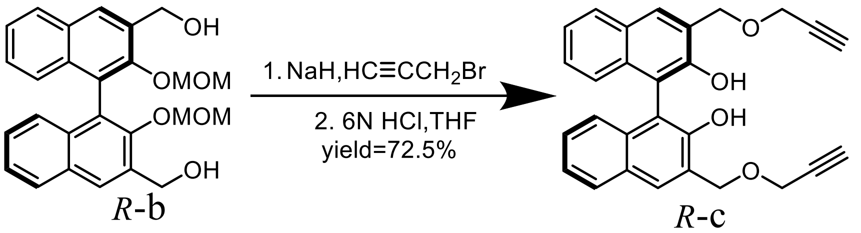

Dissolve intermediates b (2.0 g, 4.6 mmol) with 10 ml of ultra-dry THF and add to a 50 mL single-mouth flask, then add sodium hydride (0.49 g, 20.2 mmol) in batches and continue the reaction for 2 hours, then slowly add 3-bromopropyne (1.8 mL, 23.0 mmol) to the reaction system. Allow the reaction to be overnight after dropping. After the detection reaction of thin layer chromatography spot plate (TLC), the reaction was pretreated to obtain 1.8 g of yellow oily liquid, Y=76.6 %. Under the condition of turning ice bath, the product (1.8 g, 1.5 mmol) was added to a 100 mL single-mouth bottle and dissolved with 20 mL of CH_3OH / THF (CH_3OH : THF = 2:3) solution, and after ten minutes, continue to slowly add 5mL of concentrated hydrochloric acid dropwise, continue to react for three hours. After the thin-layer chromatography spot plate (TLC) detection reaction was completed, the yellow oily liquid was obtained by spinning dry through the column (unfolding agent petroleum ether : ethyl acetate = 3:1), Y=72.5 %. ^{1}H NMR (400 MHz, $CDCl_3$) δ 7.96 (s, 2H), 7.87 (d, J = 8.0 Hz, 2H), 7.35 (t, J = 7.4 Hz, 2H), 7.32 - 7.21 (m, 2H), 7.11 (d, J = 8.3 Hz, 2H), 6.01 (s, 2H), 4.96 (s, 4H), 4.35 (d, J = 2.2 Hz, 4H), 2.51 (s, 2H). ^{13}C NMR (101MHz, $CDCl_3$) δ 150.05,132.49, 128.46, 127.97, 127.29, 126.12, 124.45,123.4, 123.04, 112.11,78.25, 74.21,67.69, 56.87 (ppm).

S1.4 Synthesis of probe *R*-**1**

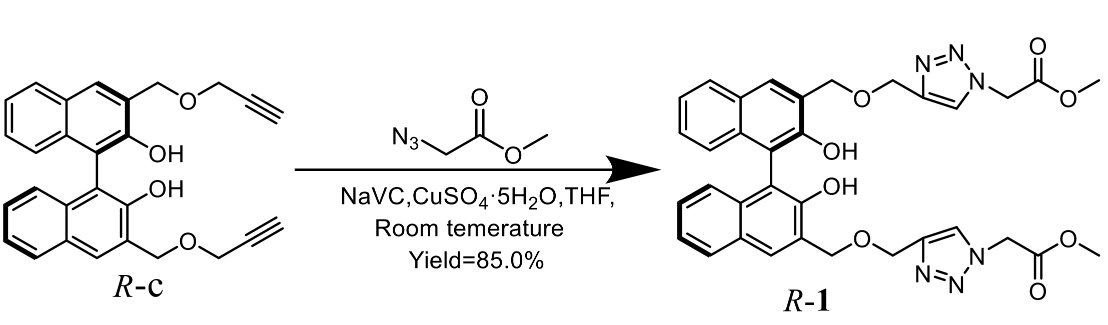

R-3,3'-bis((prop-2-yn-1-yloxy) methyl)-[1,1'-binaphthalene]-2,2'-diol (1.0 g, 2.26 mmol) and 2-azidoa- cetate(0.5 mL, 5.4 mmol) were added to a 100 mL aubergine flask with 20 ml of tetrahydrofuran. After the reaction system was stirred for 5 min at 0 °C, Sodium ascorbate(0.98 g, 4.9 mmol), copper sulfate pentahydrate (0.56 g, 2.2 mmol), and 8 mL of water were injected into the reaction system overnight. After TLC detected the complete reaction, the reaction was quenched into ice water. dichloromethane was extracted three times, washed once with saturated salt water, and dried with anhydrous magnesium sulfate. Filter and silica gel column chromatography (unfolding agent petroleum ether : ethyl acetate = 1:2) to obtain a white solid 1.25 g, Y=85.0 %. M.P. 65-67 °C. $[\alpha]_D^{25}$ 75 (c=1, CH_3OH). MS-ESI m/z: calcd for $[C_{34}H_{32}N_6O_8+H]^+$ 653.2360; found 653.2395. ^1H NMR (400 MHz, $CDCl_3$) δ 7.90 (s, 2H), 7.85 (d, *J* = 8.1 Hz, 2H), 7.69 (s, 2H), 7.33 (t, *J* = 7.4 Hz, 2H), 7.25 (dd, *J* = 15.2, 7.1 Hz, 2H), 7.10 (d, *J* = 8.4 Hz, 2H), 6.70 – 6.47 (m, 2H), 5.08 (s, 4H), 4.95 (ABq, $\Delta\delta_{AB}$=0.07, *J* = 12.5 Hz, 4H), 4.84 (s, 4H), 3.74 (s, 6H). ^{13}C NMR (101 MHz, $CDCl_3$) δ 166.65, 151.35, 145.10, 133.64, 129.33, 128.79, 128.16, 126.90, 125.76, 124.54, 124.20, 123.81, 113.83, 69.70, 63.90, 52.95, 50.62. (ppm).

S1.5 Synthesis of probe *S*-**1**

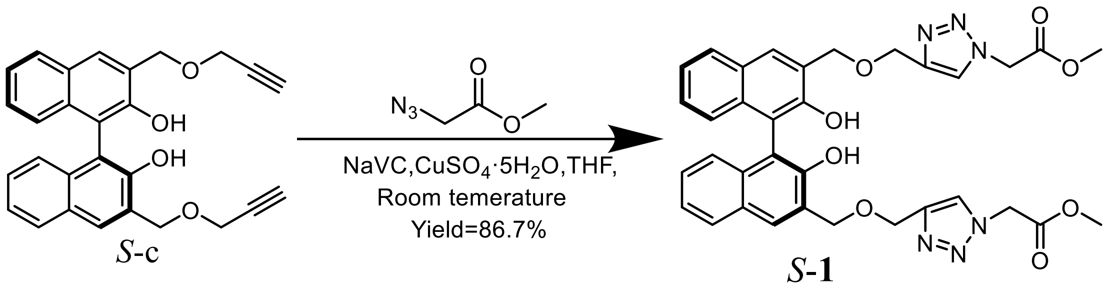

The synthesis procedure of the intermediate is the same as that of the *R* configuration.

S-3,3'-bis((prop-2-yn-1-yloxy) methyl)-[1,1'-binaphthalene]-2,2'-diol (0.30g, 0.67mmol) and 2-azidoacetate (0.16 mL, 1.60 mmol) were added to a 100 mL aubergine flask with 10 ml of tetrahydrofuran. After the reaction system was stirred for 5 min at 0 °C, Sodium ascorbate (0.30 g, 1.50 mmol), copper sulfate pentahydrate (0.17 g, 0.67mmol), and 6 mL of water were injected into the reaction system overnight. After TLC detected the complete reaction, the reaction was quenched into ice water; dichloromethane was extracted three times, washed once with saturated salt water, and dried with anhydrous magnesium sulfate. Filter and silica gel column chromatography (unfolding agent petroleum ether : ethyl acetate = 1:2) to obtain a white solid 0.38 g, Y= 86.7 %. M.P. 62-63 °C. $[\alpha]_D^{25}$-75 (c=1, CH_3OH). MS-ESI m/z: calcd for $[C_{34}H_{32}N_6O_8+H]^+$ 653.2360; found 653.2395. ^1H NMR (400 MHz, $CDCl_3$) δ 7.86 (s, 2H), 7.82 (d, J = 7.9 Hz, 2H), 7.66 (d, J = 1.8 Hz, 2H), 7.31 (t, J = 6.9 Hz, 2H), 7.22 (t, J = 7.6 Hz, 2H), 7.08 (d, J = 8.2 Hz, 2H), 6.67 (s, 2H), 5.05 (d, J = 1.9 Hz, 4H), 4.91 (ABq, $\Delta\delta_{AB}$ = 0.06, J =12.5Hz, 4H), 4.81 (s, 4H), 3.72 (d, J = 2.1 Hz, 6H). ^{13}C NMR (101 MHz, $CDCl_3$) δ 166.68, 151.34, 145.05, 133.61, 129.27, 128.75, 128.17, 126.91, 125.68, 124.54, 124.23, 123.83, 113.83, 69.74, 63.86, 53.01, 50.61. (ppm).

S2、^1H NMR, ^{13}C NMR and ESI-MS

S2.1 ^1H NMR, ^{13}C NMR of intermediate products

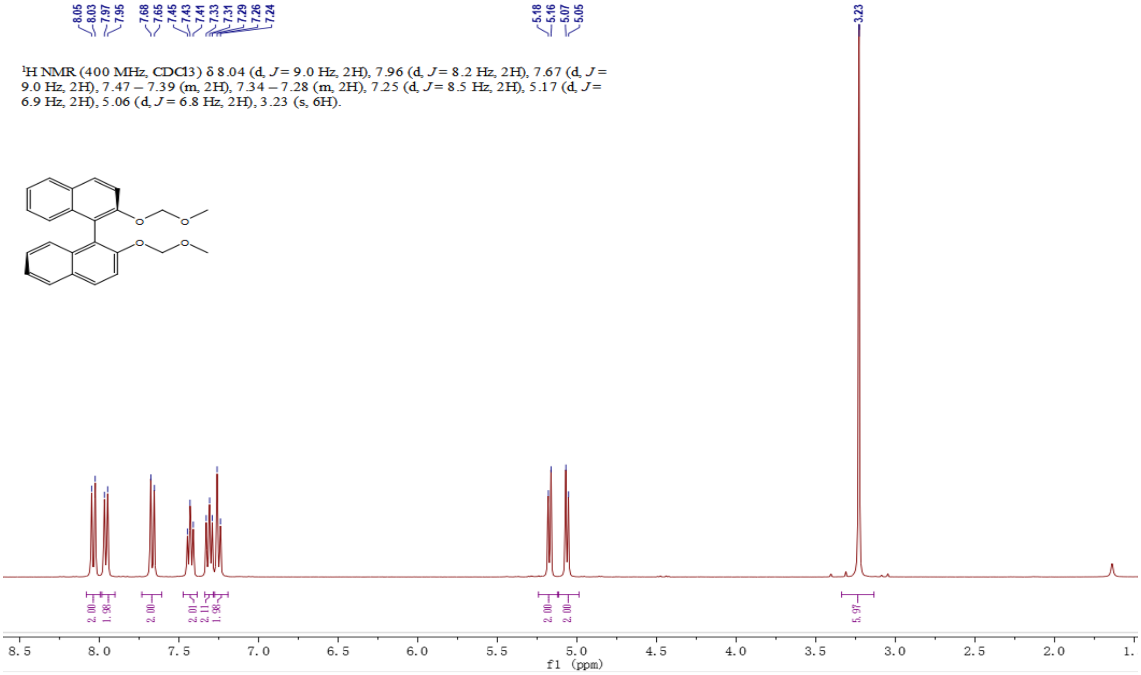

¹H NMR (400 MHz, CDCl3) δ 8.04 (d, J = 9.0 Hz, 2H), 7.96 (d, J = 8.2 Hz, 2H), 7.67 (d, J = 9.0 Hz, 2H), 7.47 – 7.39 (m, 2H), 7.34 – 7.28 (m, 2H), 7.25 (d, J = 8.5 Hz, 2H), 5.17 (d, J = 6.9 Hz, 2H), 5.06 (d, J = 6.8 Hz, 2H), 3.23 (s, 6H).
f1 (ppm)

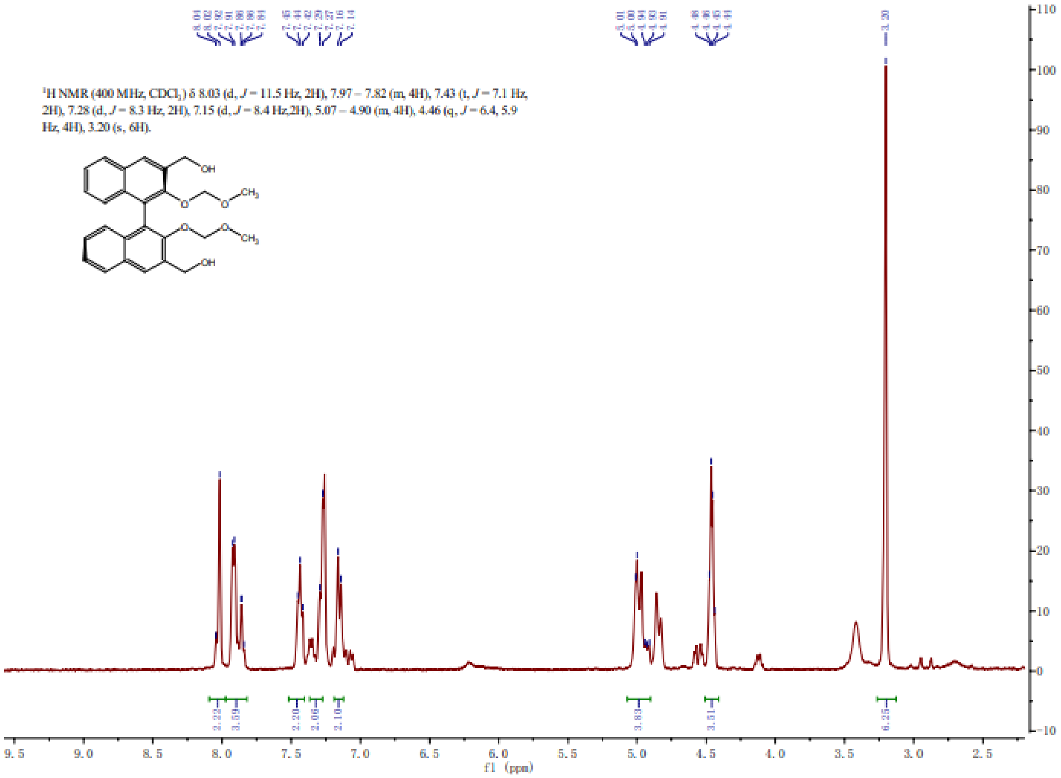

¹H NMR (400 MHz, CDCl3) δ 8.03 (d, J = 11.5 Hz, 2H), 7.97 – 7.82 (m, 4H), 7.43 (t, J = 7.1 Hz, 2H), 7.28 (d, J = 8.3 Hz, 2H), 7.15 (d, J = 8.4 Hz,2H), 5.07 – 4.90 (m, 4H), 4.46 (q, J = 6.4, 5.9 Hz, 4H), 3.20 (s, 6H).
OH
CH3
f1 (ppm)

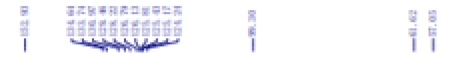

^{13}C NMR (101 MHz, $CDCl_3$) δ 152.93, 134.64, 133.74, 130.97, 129.46, 128.22, 126.79, 126.13, 125.81, 125.43, 125.17, 99.30, 61.62, 57.05.

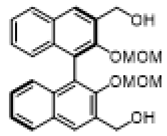

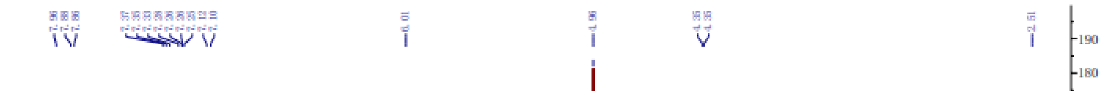

^{1}H NMR (400 MHz, $CDCl_3$) δ 7.96 (s, 2H), 7.87 (d, J = 8.0 Hz, 2H), 7.35 (t, J = 7.4 Hz, 2H), 7.32 – 7.21 (m, 2H), 7.11 (d, J = 8.3 Hz, 2H), 6.01 (s, 2H), 4.96 (s, 4H), 4.35 (d, J = 2.2 Hz, 4H), 2.51 (s, 2H).

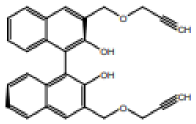

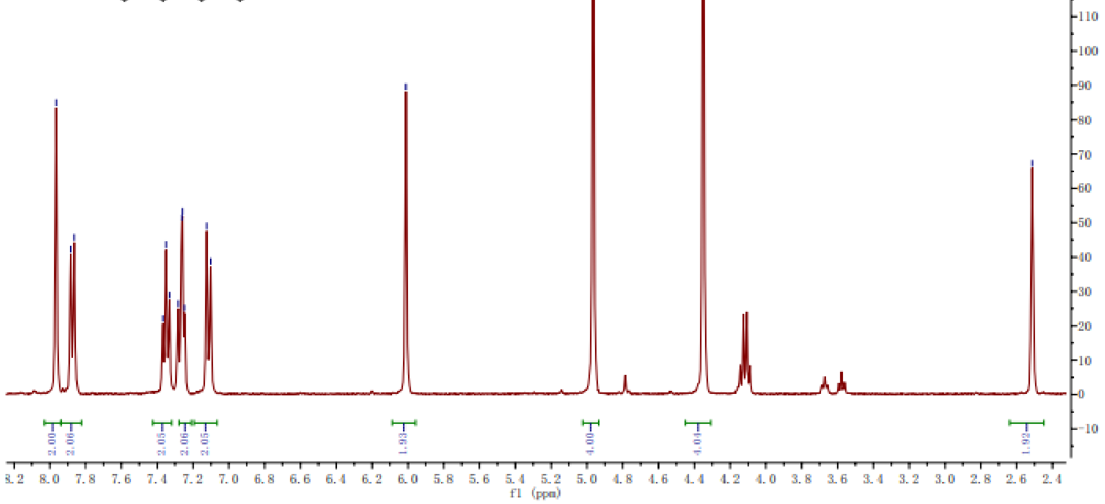

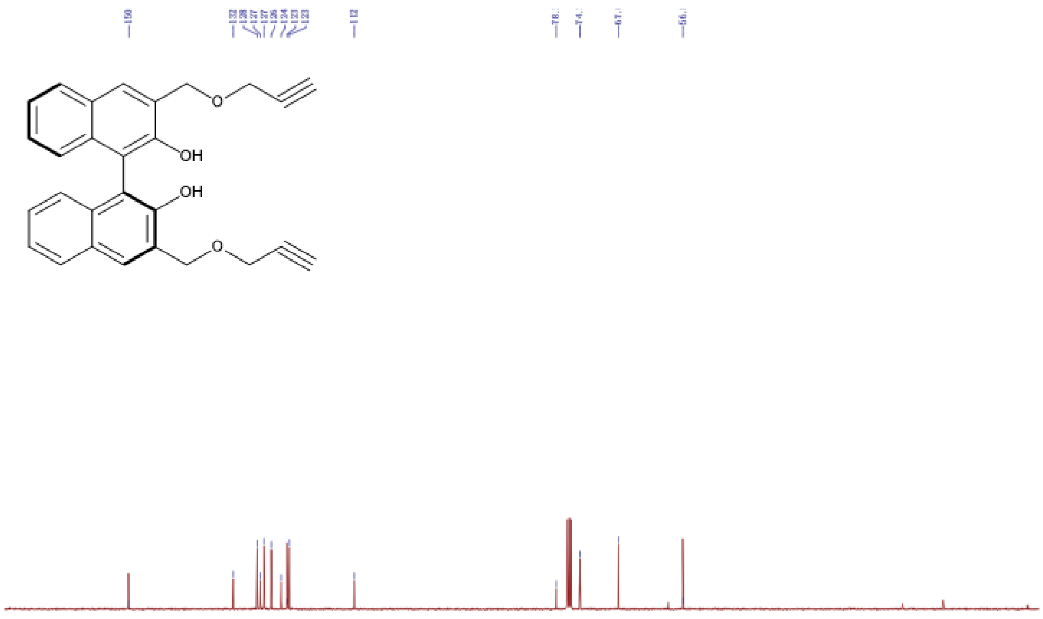

S2.2 ^{1}H NMR, ^{13}C NMR of *R*-**1**

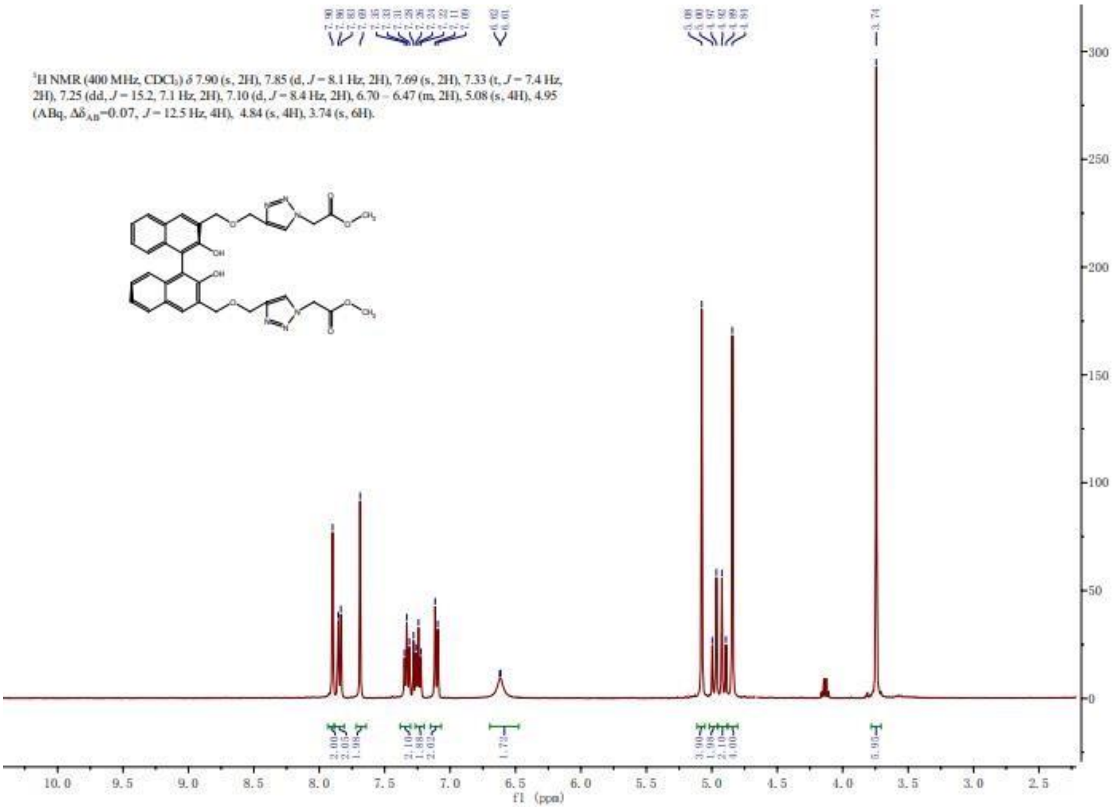

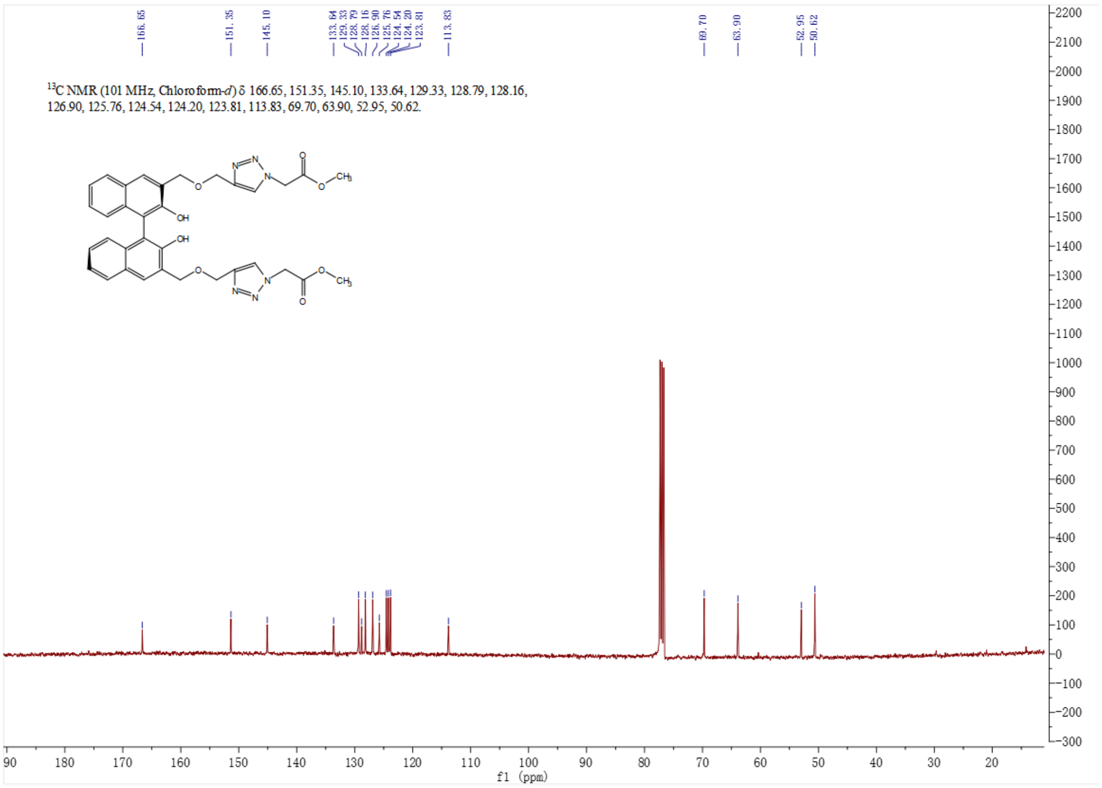

S2.3 ^{1}H NMR, ^{13}C NMR of *S*-**1**

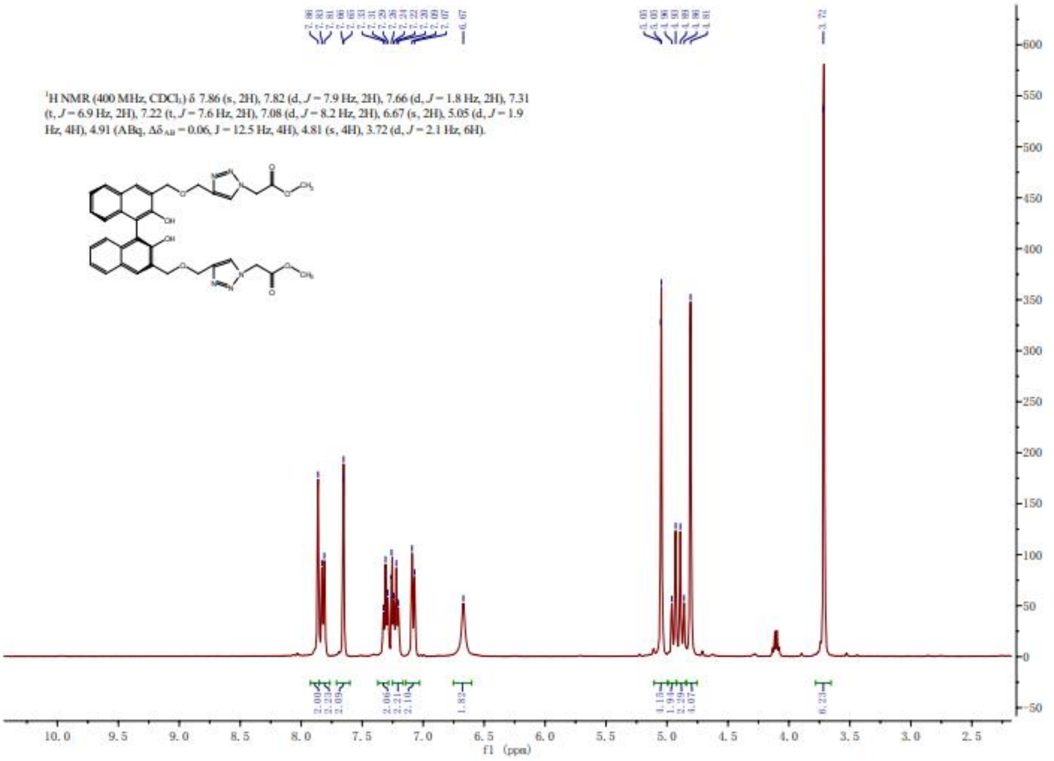

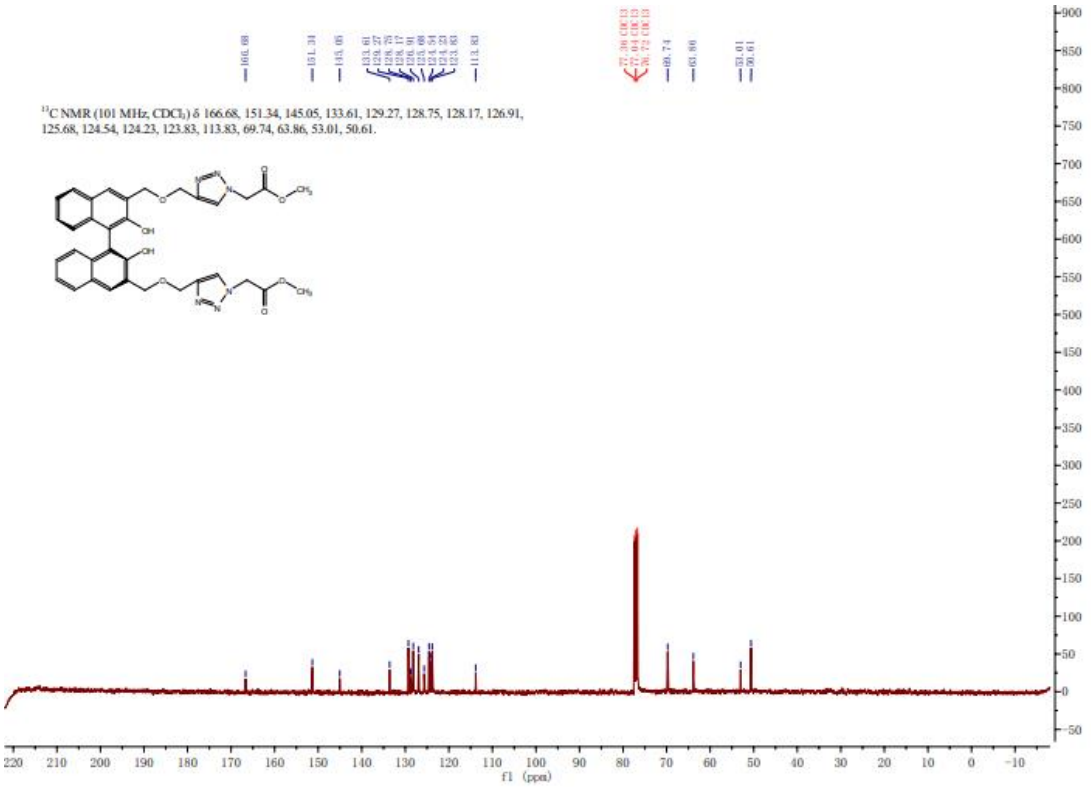

S2.4 MS-ESI of *R*-**1**

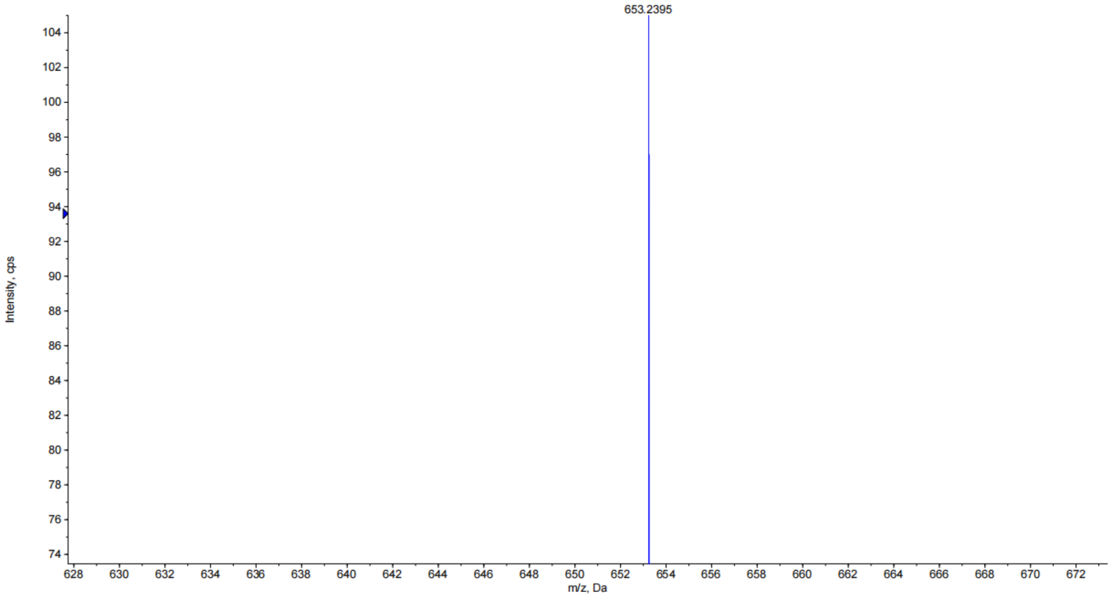

S2.5 MS-ESI of *S*-1

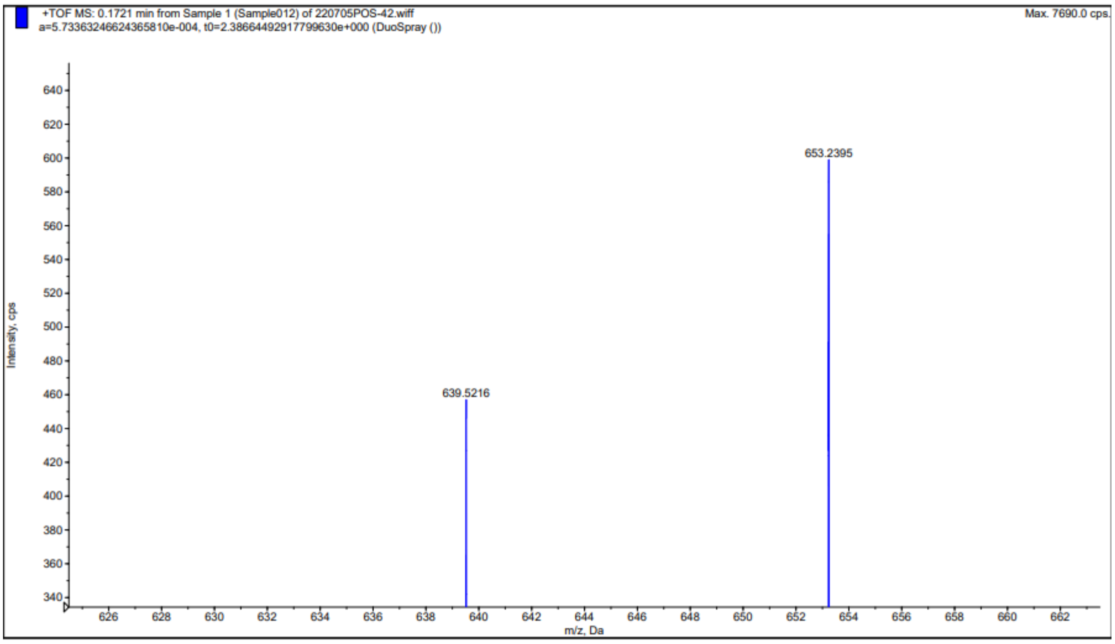

S3、Fluorescence spectra

S3.1 Fluorescence spectra of *S*-**1**

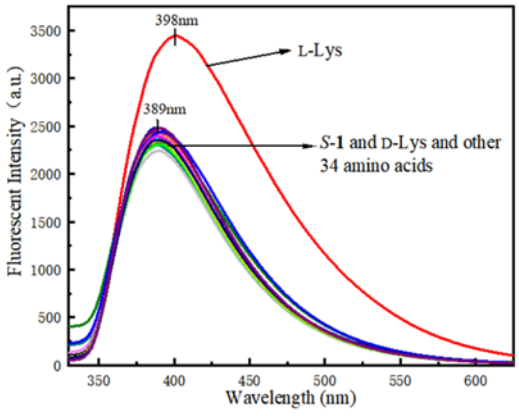

(a)

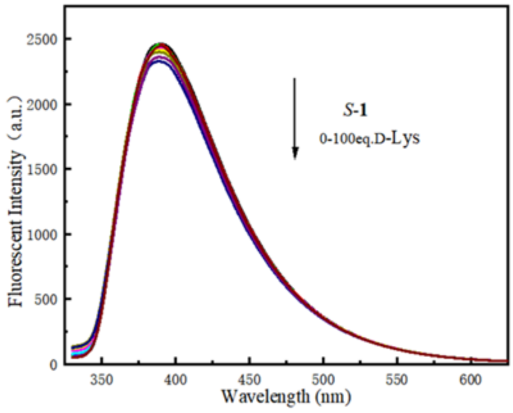

(b)

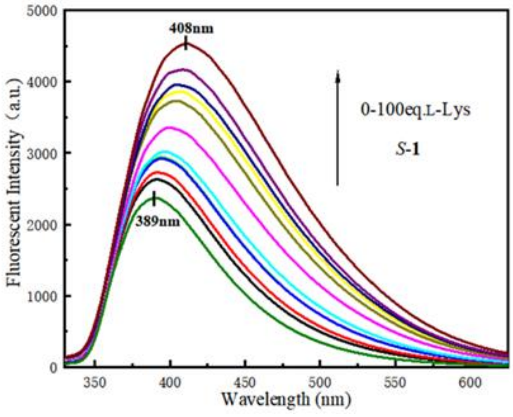

(c)

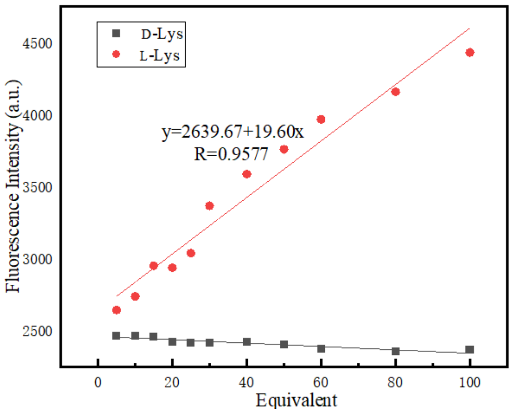

(d)

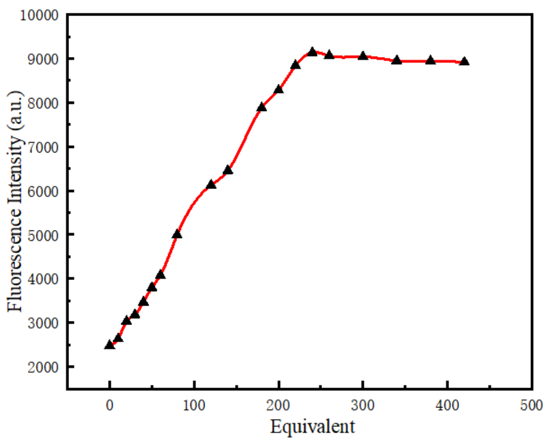

(e)

Figure S1: (a) Fluorescence spectra of *S*-**1** (2.0×10^{-5} M in CH_3OH) with thirty-six ordinary amino acids (20.0equivalents) (λ_{ex}=318 nm, slits=5.0/2.5nm). (b) Fluorescence titration of *S*-**1** with D-Lysine in CH_3OH. (c) Fluorescence titration of *S*-**1** with L-Lysine CH_3OH. (d) Fluorescence intensities at λ=389 nm versus the equivalents of lysine (λ_{ex}=318 nm, slits=5.0/2.5 nm) (e) Fluorescence intensity against L-Lys concentration (λ=318 nm). The association constant (K_a) of *S*-**1** and L-lys was calculated to be 7.85×10^{6} L•mol $^{-1}$.

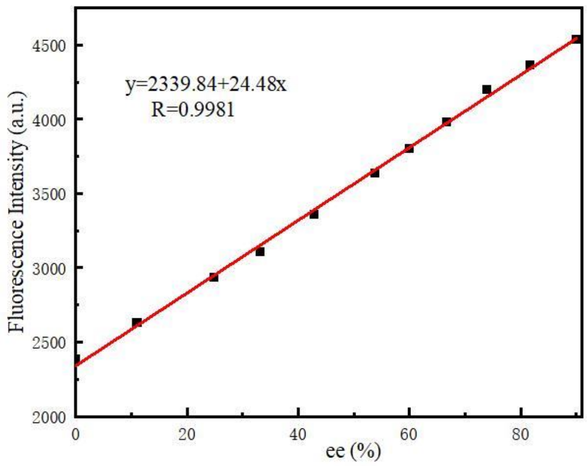

Figure S2: Fluorescence response at λ_{ex} =389nm for the mixtures of *S*-**1** toward L-lysine (100.0 equiv.) at various ee values.

S4、UV-vis titration

Probes *R*-**1** and *S*-**1** were UV absorption titration with L-Lys and D-Lys. From the UV absorption spectrum, we found that the change in wavelength at 250-350 nm is relatively insignificant, so speculate that this absorption peak is a л-л conjugate that occurs on the naphthalene. With the increasing concentration of L-Lys, the absorption of *R*-**1** and *S*-**1** at 230 nm gradually increases, we speculate that the change of this absorption peak is *л*-*л* conjugation that occurs on the triazole group, so its absorption also increases with the increase of concentration, which further confirms that our core force mainly occurs on the triazole group.

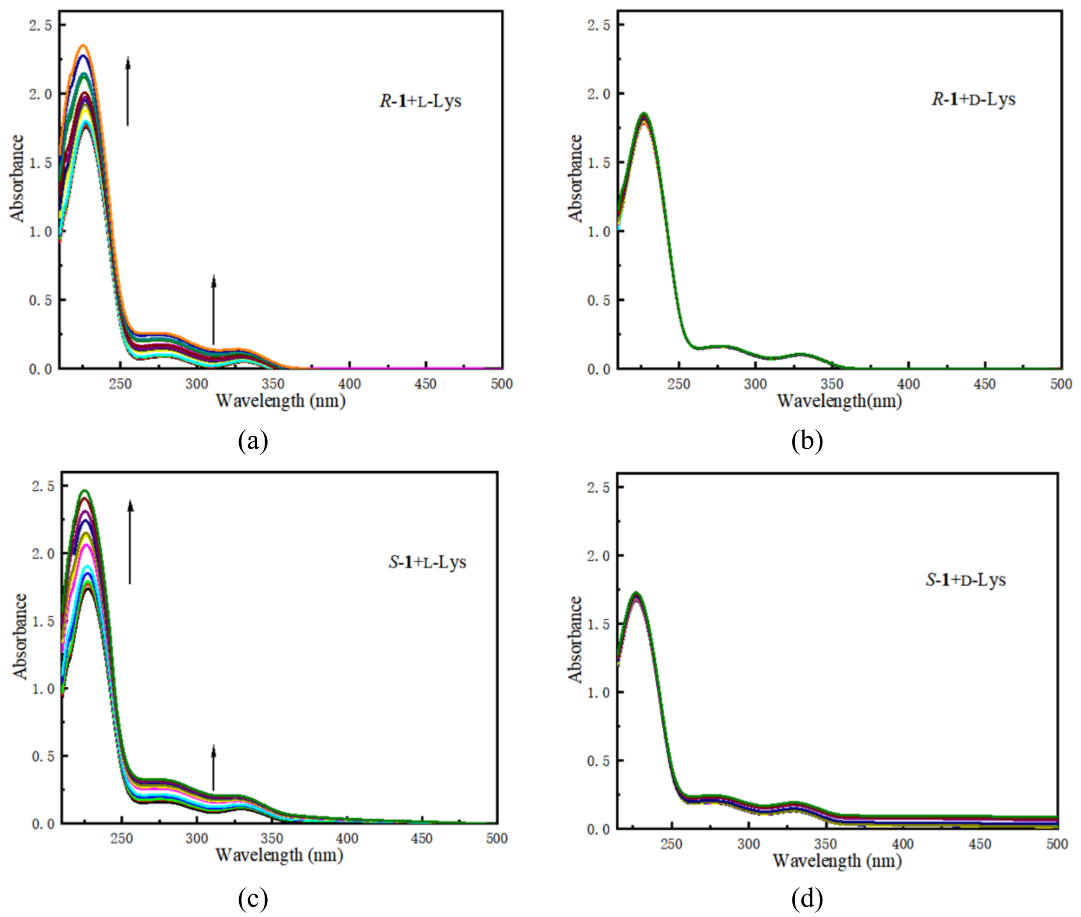

Figure S3: (a) UV absorption titration spectra of L-Lys to probe *R*-**1**. (b) UV absorption titration spectra of D-Lys to probe *R*-**1**. (c) UV absorption titration spectra of L-Lys to probe *S*-**1**. (d) UV absorption titration spectra of D-Lys to probe *S*-**1**. (0-100eq.)
